# The Pay System in Hospitals

**Published:** 1897-05-01

**Authors:** 


### THE PAY SYSTEM IN HOSPITALS.

In the *Charity Organisation Review* there is an article by Mr. Timothy Holmes on the pay system in hospitals, in which he makes many statements with which we think most people who have studied this question will, with certain qualifications, cordially agree. His conclusions are as follows:—

"1. Institutions are necessary in large towns where patients can be received for medical or surgical treatment on payment."—Certainly.

"2. These institutions should be separate from the public voluntary hospitals."—Again, we can agree with Mr. Holmes so far as the proposition concerns those who are able to pay the entire expenses of board, lodging, and medical and surgical attendance at such institutions, and so far as it concerns towns which are properly supplied with nursing homes or pay hospitals. It is obvious, however, that in regard to this matter the same rule cannot be made to apply to all places, and that the question whether or not to admit a given individual, whatever his circumstances, as a paying patient must always be complicated by the alternative question, What will happen to him if he is not admitted?

"3. They should be managed on commercial principles, so as to secure an adequate return on the capital employed, and an adequate remuneration to the medical attendants."—Undoubtedly.

"4. The reception of paying out-patients at general or special hospitals is undesirable."—As a broad principle, and so far as concerns the older conceptions of hospital work, this proposition also may be accepted. But it must be remembered that in the older conceptions of hospital work the special hospital had no place; and, although we are quite ready to admit that when the time shall arrive when the provident dispensary system shall have become thoroughly established —so thoroughly established that the members of these institutions shall in return for their provident payments have access to special, as well as to general medical advice—there will be no excuse for taking fees at special hospitals, we cannot help seeing that such time has not yet arrived. So long as specialists maintain that they cannot afford to give their services for less than consulting fees, a matter as to which we venture to have grave doubts, it is quite clear that a very large number of people must either go without special help, or must sponge upon the hospitals, a thing that they have hitherto shown no great backwardness in doing, or must be made to pay for their treatment at the special hospitals. General hospitals are another matter. Their general out-patient departments should be strictly kept for those who are not able to pay and for those introduced for consultative or other purposes by medical practitioners.

"5. Provident dispensaries properly organised and well managed would be most useful under a reformed out-patient system; but it is very doubtful whether such institutions ought to be incorporated with general hospitals."—We would go further, and say that the success of provident dispensaries depends largely upon their not being incorporated with the hospitals. Nevertheless they should either provide their clients with special advice or make arrangements with the special institutions for the purpose, and it may properly be urged that the medical officers of provident dispensaries should have some sort of assurance that the cases which they send to the hospitals either for consultative purposes or for in-patient treatment should have some definite claim upon the hospital authorities.

So far, as we have indicated above, we can heartily agree with Mr. Holmes, as also with all that he says as to the disadvantages of State support, and with many of his remarks about the anomalous position in which the pay question now stands at the various hospitals.

But we cannot help feeling that Mr. Holmes fails, in his paper, to show a full appreciation of the complexity of the problem which faces hospital managers in regard to accepting, and still more in regard to demanding, payment from hospital patients. Speaking of blending the pay and the gratuitous systems in in-patient practice, he says: "We are told in general terms that patients pay willingly, and the hospital finances benefit. No doubt a man would be willing enough to buy a piece of bread and cheese on the terms of paying for the bread only; and in the same way a man who has medical treatment and maintenance in a hospital may be assumed to be willing to purchase them by paying for the maintenance only—but is it honest? It hardly seems so." This does not seem to us quite fair. We do not here allude to the question of pay wards, but to the broad principle of accepting payment from patients in voluntary hospitals; and it seems to us that Mr. Holmes, while laying down propositions as to extreme cases with which many people will agree, fails entirely to give an answer to the real question which, as things exist at the present time, hospital managers have to answer every week, viz., Shall we ask this poor fellow to pay something towards his support or even towards his treatment, although we know that he cannot afford to pay for it altogether; or shall we take him in free gratis and for nothing, at the expense of the charity, although we know perfectly well that he can pay a fairly substantial sum towards his support, and although, as a fact, he does not even ask to be admitted for nothing; or, as a third resource, shall we refuse to have anything to do with him because he is not a "real object of charity," and would obviously find no great difficulty in paying his half-crown to his doctor at home?

This is the question which is constantly occurring to hospital managers, and which they have to solve in accordance with the prevailing feeling of their committee and the prevailing tradition at their hospital. Hence, on the one hand, a vast waste of funds which might well be employed in the treatment of those who are entirely helpless; and, on the other hand, an occasional hardship where a poor man is refused admission because he is not poor enough.

Now to this question, which at the present moment is the one of most immediate urgency, Mr. Holmes gives no answer; nor does anyone who remains blind to the fact that between the mass who can pay all "on commercial principles" and the mass who can pay nothing, there stands a great crowd of people who can pay at least something.

Now what we maintain is that the hospitals not only are doing right in accepting the little that these people are able to give, but that are doing wrong if they refuse to ask for such contributions as such patients on the borderland are able to afford. If it were true that the world were peopled by two classes only, those sunk in poverty and those rolling in wealth, the matter would be simple. But it is not true. There is an enormous middle and lower middle class who are perfectly well able to pay for ordinary medical attendance, and who by no possible stretch of language can be called "objects of charity" even when their illness requires something more than ordinary attendance. They are even then perfectly able to pay something but not all; and we believe that the hospitals are doing a better work by accepting and even demanding that "something," little as it may be, than they would be doing were they either to refuse the patient as not being a fit object of charity, or were to accept him out-and-out as a charity; and thus, while curing him of his bodily disease, were to infect him with that terrible mental infirmity, a willingness to depend in emergencies on eleemosynary help.

As to what is to be done with the money when it is received we need not here say much, as we have already given our opinion. Broadly, however, it may be laid down that so long as the money paid by the patient does not entirely cover the cost of board, lodging, and nursing, the patient is receiving charity from the subscribers to the hospital; and, under those circumstances, the medical officers, according to the present custom of accepting "honorary" appointments, may properly give their services, and thus become participators in the good work of the institution with which they are associated. But directly the payment goes beyond this (although it may still be less than would be charged by nursing homes as they exist at present), a very substantial proportion of the sum received should go to the medical staff. We do not say that the staff would always be willing to receive it; but as a matter of common justice, first it ought to be demanded from the patients, so far as their means will go, so as to prevent any unfair competition between the "charity" and the "commercial" hospitals, and secondly, a proper share of it ought to go to the medical men who do the work. Probably Harley Street might refuse to put such money in its pocket. But there are old and feeble and helpless members of the medical profession, who are far more worthy objects of the charity of medical men than are many of the occupants of our hospital beds, and it would not be an ungraceful act if those who are in the heyday of their success were to give to such institutions as the Royal Medical Benevolent Fund such moneys as might come to them in the course of their hospital work.

